# Ethical attitudes and perspectives of AI use in medicine between Croatian and Slovenian faculty members of school of medicine: Cross-sectional study

**DOI:** 10.1371/journal.pone.0310599

**Published:** 2024-12-05

**Authors:** Štefan Grosek, Stjepan Štivić, Ana Borovečki, Marko Ćurković, Jaro Lajovic, Ana Marušić, Antonija Mijatović, Mirjana Miksić, Suzana Mimica, Eva Škrlep, Kristina Lah Tomulić, Vanja Erčulj

**Affiliations:** 1 Neonatology Section, Department of Perinatology, Division of Gynaecology and Obstetrics, University Medical Centre, Ljubljana, Ljubljana, Slovenia; 2 Institute of Bioethics, Faculty of Theology, University of Ljubljana, Ljubljana Slovenia; 3 School of Medicine, ‘A, Štampar’ School of Public Health, University of Zagreb, Zagreb, Croatia; 4 University Psychiatric Hospital Vrapče, Zagreb, Croatia; 5 Rho Sigma Research & Consulting, Ljubljana, Slovenia; 6 Center for Evidence-based Medicine, Department of Research in Biomedicine and Health, School of Medicine, University of Split, Split, Croatia; 7 University Medical Centre Maribor, Clinic for Gynecology and Perinatology, Maribor, Slovenia; 8 University Hospital Centre Osijek, Osijek, Croatia; 9 Faculty of Medicine, University of Ljubljana, Ljubljana, Slovenia; 10 Department of Pediatrics, Faculty of Medicine, University of Rijeka, Croatia Pediatric Intensive Care Unit, Department of Pediatrics, Clinical Hospital Centre Rijeka, Rijeka, Croatia; 11 Faculty of Criminal Justice and Security, University of Maribor, Ljubljana, Slovenia; Ruđer Bošković Institute, CROATIA

## Abstract

**Background:**

Artificial intelligence (AI) is present in preclinical, clinical and research work, in various branches of medicine. Researchers and teachers at school of medicines may have different ethical attitudes and perspectives about the implementation of AI systems in medicine.

**Methods:**

We conducted an online survey among researchers and teachers (RTs) at the departments and institutes of two Slovenian and four Croatian Schools of Medicine.

**Results:**

The sample included 165 and 214 researchers and teachers in Slovenia and Croatia, respectively. The sample of respondents in Slovenia and Croatia was comparable in demographical characteristics. All participants reported high emphasis on the bioethical principles when using artificial intelligence in medicine, its usefulness in certain circumstances, but also caution regarding companies providing AI systems and tools. Slovenian and Croatian researchers and teachers shared three similar perspectives on the use of AI in medicine–complying with highest ethical principles, explainability and transparency and usefulness of AI tools. Higher caution towards use of AI in medicine and effect on autonomy of physicians was expressed in Croatia, while in Slovenia high emphasis was put on understanding how AI works, but also the concerns regarding willingness and time of physicians to learn about AI.

**Conclusion:**

Slovenian and Croatian researchers and teachers share ethical attitudes and perspectives with international researchers and physicians. It is important to facilitate understanding of the implications of AI use in medicine and set a solid evidence-based ground to tackle ethical and legal issues.

## Introduction

Artificial Intelligence (AI) is breakthrough and central-role technology in the broader scope of the convergence of technologies such as nanotechnologies, information technologies, biotechnologies, cognitive technologies etc. [1]. It has been already very widely implemented in various branches of human life, including medicine [2]. China recently reported opening the first in the world AI hospital with virtual doctors, which will bring completely new dimensions of ethical issues raised by AI systems incorporated in the healthcare system i.e. in the relation between virtual doctor and patient [3]. Medicine as we know from yesterday transformed with AI into modern medicine today [4, 5]. Medical bioethics which was well defined concept [6] had to react quickly and grow with the development of AI involvement in medicine, not only reshaping relations between physicians and patients but also between physicians and AI systems which offer completely new possibilities of diagnostic and treatment options [7, 8]. Thus, today, in addition to the well-known principles, there is consideration of those that would cover a much wider scope of related issues, including privacy and explainability of AI in medicine [9]. We now have a large number scientific and popular papers on AI use along with problem-solving ideas, in order to grapple with ethical issues in medicine and healthcare.

Many relevant institutions, such as the European Council, and various international committees took interest in AI use in medicine and fields dealing with regulations concerning ethical and legal issues. Over the past decade, the medical community has increasingly recognized the significance of ethics in AI, drawing considerable interest from researchers. For example, a Medline search with key-words “artificial intelligence”, “ethics” bring in the last 10 years 1816 articles, while in the previous 40 years only 50 articles. According to some research, the most affected physicians regarding AI ethics are radiologists, geneticists, ophthalmologists, and dermatologists [10, 11] of which the least concerned are ophthalmologists and dermatologists [12]. However, a concern in one area does not necessarily exclude a positive attitude towards the benefits in diagnostic processes.

Ethical attitudes on AI use in medicine oscillate between warnings of unsafety and optimism, often intertwined. The optimistic attitude believers think that AI can personalize medicine, enhance the patient-physician relationship, and improve outcomes by providing more precise treatment [13–15]. At the same time, multiple concerns emerge among patients related to the safety of AI, threats to patient autonomy, potential increases in healthcare costs, data-source bias, and data security [16]. There are also concerns about the reliability of data used to train AI algorithms [17–19] and the potential for AI to perpetuate existing biases [15], making incorrect diagnoses [14] and the change of the status of physician in that context. However, since the beginning there have been prudent approaches in relevant documents that require legal regulation and ethical discussion.

There is some evidence on the ethical perspectives and legal solutions for the use of AI in medicine. Richardson et al. [16] conducted survey study on patients’ apprehension of AI in medicine, and Alanazi’s [17] quantitative study identified the crucial areas of using AI in medicine, highlighting the categories of safety, responsible, and transparent AI systems as crucial for patients. In Japan, Katirai et al. [20] discussed the perspectives of patients and the public regarding the use of AI expecting improvement in medical service, while also caring about autonomy, accountability, and inequalities. Busch et al.’s [21] study explored medical students’ attitudes towards AI in medical education, and Weidener & Fischer [22] study on students’ perceptions of AI and ethical implications, emphasized the lack of AI education in curricula and the need for the introduction of AI in education. Civaner et. al. [23] conducted a study among medical students on the positive and negative sides of AI and stressed the need for equipping future physicians with the knowledge and skills to effectively use AI. In a recent study by Kim Y. J. et al. [24], they identified five crucial ethical domains—accountability, fairness, fitness for purpose, reliability and validity, and transparency—across the AI lifecycle when developing solutions to assess and reduce the risk of AI exacerbating health inequities. Based on these domains and involving relevant stakeholders, they believe it is possible to ensure an equitable application of AI in healthcare. In the study by Martinho et al. [25], the attitudes of medical doctors on the ethics of AI in medicine were explored. Among 353 statements gathered after reviewing the literature, the authors categorized them into fifteen topics related to the AI ethics and designed a 40-item questionnaire. They discussed the main perspectives on the ethics of AI in medicine, gathered from a survey of medical doctors in the Netherlands, Portugal, and the U.S. These perspectives reflect the views on the implementation of AI into medical practices and its ethical implications. These are: 1) AI is a Helpful Tool: Let Physicians Do What They Were Trained For—emphasizes efficiency and automation provided by AI, which will allow doctors to expanding their knowledge and skills; 2) Rules & Regulations are Crucial: Private Companies Only Think About Money—shows a distrust in private tech companies and underlines the need that AI systems in medicine are implemented ethically; 3) Ethics is Enough: Private Companies Can Be Trusted—suggests that ethical guidelines are sufficient to ensure that tech companies operate in the best interest of healthcare; 4) Explainable AI Tools: Learning is Necessary and Inevitable—highlights the importance of explainability in AI tools, ensuring that doctors are part in the AI systems development.

In our preliminary study (unpublished), which served as a pilot study, among researchers and teachers (RTs) in School of Medicine in Ljubljana we followed Marthino et al. research approach and adopted their questionnaire. Preliminary results were comparable with Martinho‘s et al. [25] study. Furthermore, they showed that RTs are interested in learning about AI and that RTs should be involved in AI implementation in medicine and that responsibility for AI’s failures should be shared between the users and developers [26]. After this pilot study we extended our research and surveyed Slovenian and Croatian RTs working at two Slovenian and four Croatian School of Medicine. Our primary objectives were to explore and compare attitudes on ethics of AI in medicine between the two countries and to delineate main perspectives on ethics of AI in medicine among RTs in Slovenia and Croatia.

## Methods

### Study design and target population

A cross-sectional study was carried out in Slovenia and Croatia in 2023. An online survey was conducted among researchers and teachers at medical schools. The link with the invitation to participate in the study was sent to all researchers and teachers employed in the departments and institutes at two Slovenian and four Croatian Faculties or Schools of Medicine (Faculty of Medicine, University of Ljubljana; Faculty of Medicine, University of Maribor; School of Medicine, University of Zagreb; School of Medicine, University of Split; Faculty of Medicine, University of Rijeka; Faculty of Medicine, University of Osijek). Two reminders were sent to facilitate the participation in the study and increase the response rate. In Slovenia the data was collected between 15 May 2023 and 12 June 2023 in Ljubljana and between 12 October and 7 December 2023 in Maribor, and in Croatia between 16 October and 27 November 2023. The link with the invitation to participate in the study was sent through the dean’s office via e-mail to 1764 (Slovenia 536, Croatia 1228) RTs which were employed at the time. Ethical approval was obtained from the Medical Ethics Committee of Republic Slovenia (no. of approval: 0120-87/2023/6; October 5, 2023) and from ethical commissions of 4 faculties of medicine in Croatia (Zagreb no. 641-01/23-01/01; September 20, 2023; Split no. 2181-198-03-04-23-0069; September 27, 2023; Rijeka no. 2170-1-42-04-36/1-23-7; September 29, 2023; Osijek 602-06/23-08/03; September 18, 2023). The participation in the study was voluntary and anonymous–researchers willing to participate in the study followed the invitation link and by doing so agreed to participate in the anonymous study. Additional written informed consent was deemed unnecessary.

### Sampling method

The study aimed to include all RTs employed in the departments and institutes at two Slovenian and four Croatian National Schools of Medicine, as survey participants. The link with the invitation to participate in the study was sent via e-mail to 1764 (Slovenia 536, Croatia 1228) RTs which were employed at the time.

### Data collection tool

From the authors Martinho et al. [25] we obtained written permission on December 5, 2022, to translate, adopt and use their questionnaire on ethics surrounding health AI in our study (S1 Appendix). The questionnaire included 40 items to which respondents replied on a five-point Likert scale of agreement (from completely disagree to completely agree).

The questionnaire was localized to Slovenian and Croatian environment [27]. Two independent translators translated the questionnaire into Slovenian and Croatian language, compared the statements and resolved the discrepancies. After reaching consensus, the questionnaire was back-translated to English and the meaning of the items was investigated. The final version of the questionnaire was obtained. Cognitive interviews with 28 researchers, teachers and/or physicians were performed to carefully examine the meaning of each statement and further improve the understanding of the statements and evoke the same interpretation of the statement in all respondents. The extra statement was added to determine the preliminary status of AI in medicine (“Artificial intelligence is influencing decision-making in clinical and preclinical medicine”). The section on demographic information included age, gender, length of professional experience, and field of activity, where they could choose from options (a) clinical practice, (b) preclinical practice, and (c) other. In the invitation letter we specifically asked addressees to participate in the survey on the ethical aspects of the use of AI in medicine and healthcare. Final questionnaires, used in Slovenia and Croatia are provided in S2 Appendix and S3 Appendix, respectively.

### Statistics

Categorical variables were described by frequencies and percentages, non-normally distributed continuous variables by medians and interquartile ranges. All respondents did not provide answer to all questions–all the answers provided were included in the analysis. Comparison between the two countries in the sample characteristics were performed using chi-square test for categorical and Mann-Whitney U test for continuous variables. Prior the analysis, values of each statement, measured on a five-point Likert scale of agreement, were recoded into two categories. The first included the answers from 1 to 3 indicating lower agreement with the statement and the second the answers 4 and 5 indicating higher agreement with the statement. The association between each recoded statement and country was examined using univariate logistic regression analysis.

To examine the perspectives of researchers and teachers, principal component analysis with the orthogonal rotation was performed on the original items about the use of AI in medicine. Principal component analysis reduces the number of variables into components–linear combinations of the measured variables that maximally explain the variance of the variables. It is used to identify patterns in the data. To distinguish between perceptions of the RTs in the two countries, principal component analysis was performed separately for each country. The number of components extracted was based on the examination of the scree diagram [28], the number of items with high loading on each component and component interpretability. Furthermore, the broken stick method [29] was considered in which the eigenvalue of a given component is compared to the value of the one that would be expected by chance (if the total variance would be distributed equally among the components). Components with higher eigenvalue after varimax rotation than generated by the broken stick model were retained. The names of each component were based on the original research of Martinho et al. [25]. In their research they predefined ethic clusters “compiled from 22 major guidelines of AI ethics as a guidance tool” (pp.3). Items of the questionnaire (S1 Appendix) were assigned to clusters Privacy (statements 1–4), Fairness (5–8); Accountability (9–10,40); Transparency (11); Safety and cybersecurity (12–13,39); Human Oversight (18); Explainability (15–17); Future of Employment (19–20, 22); Responsible Research Funding (23–24); Education About AI (25,34); Human Autonomy (18); Certification of AI products (29–30); Ethical Design (31− 33); Health specific deliberations (14,21,26–27,36–38); and AI in the Covid-19 pandemic (28,35). Our components were named after the most prevalent statements from Martinho et al. [25] clusters with highest weights on each component. All statistical testing was performed at the significance level α = 0.05. No correction for multiple testing was applied. Program IBM SPSS, version 28 was used for the statistical analysis.

## Results

The response rate in Slovenia and Croatia was 28% and 15%, respectively. In Slovenia and Croatia, the sample included 165 and 214 RTs, respectively, but all respondents did not provide demographical information. The latter was available for 150 Slovene and 186 Croatian RTs. The sample description of RTs who provided the demographical data is provided in **Table 1**. The two groups of RTs were comparable in gender (p = 0.738), age (p = 0.150), working experience (p = 0.166) and working position (p = 0.283) (**Table 1**). Croatian sample included 79 males (42.5%), 55 (29.7%) working in preclinical and 106 (57.3%) in clinical practice. Half of the Croatian RTs included in the research were 50 years old or older (IQR: 40–58). Half of them had 23 years of working experience or more (IQR: 15–33). Slovenian sample included 61 males (40.7%), 37 (24.8%) RTs working in preclinical and 84 (56.4%) in clinical practice. Half of the RTs included in the research were 49 years old or older (IQR: 36–55). Half of them had 25 years of working experience or more (IQR: 10–30).

**Table 1:** Sample description.

	CROATIA	SLOVENIA	P
	(n = 186)	(n = 150)	
Male gender	79 (42.5)	61 (40.7)	0.738
Median (IQR; n) age	50 (40–58; 186)	49 (36–55; 149)	0.166
Median (IQR; n) working experience	23 (15–33; 185)	22.5 (10–30; 148)	0.150
Working position			0.283
Preclinical	55 (29.7)	37 (24.8)	
Clinical	106 (57.3)	84 (56.4)	
Other	24 (13)	28 (18.8)	

IQR-interquartile range

**Table 2** includes statements with which the highest share (> 60%) of RTs agreed or strongly agreed in at least one country. The top three statements pertain to medical ethics and clear rules–more than 90% of respondents in each country agree or strongly agree that AI in medicine should be used and developed with consideration to ethical and bioethical principles. It should be used with clear rules and RTs should be included in the development of AI tools. RTs from both countries believe that AI healthcare tools should be tested in randomized clinical trials. No statistically significant differences in agreement with the described statements between the countries existed.

**Table 2:** Statements with highest agreement^a^ per country and results of univariate logistic regression (data shown as frequencies (percentages); number of respondents; OR = odds ratio; CI = confidence interval).

	Croatia (n = 214)	Slovenia (n = 165)	OR (95% CI)	P
(3) Without clear rules about data usage, storage, and anonymization, AI should never be used in healthcare.	193 (90.2); 214	151 (91.5); 165	1.17 (0.58–2.38)	0.658
(9) AI developers must be bound by medical ethics.	193 (93.7); 206	149 (92); 162	0.77 (0.35–1.71)	0.525
(32) Healthcare AI technology must be aligned with bioethical principles.	182 (95.3); 191	142 (94); 151	0.78 (0.3–2.02)	0.608
(33) Medical doctors must participate in the design process of AI for healthcare.	175 (91.6); 191	142 (94); 151	1.44 (0.62–3.36)	0.396
(30) AI healthcare products must be tested in randomized clinical trials, which is the strongest source of medical evidence.	172 (89.1); 193	131 (85.6); 153	0.73 (0.38–1.38)	0.329
(8) We should be conservative in promoting AI in healthcare because of the unresolved ethical issues.	167 (81.1); 206	92 (56.8); 162	0.31 (0.19–0.49)	< 0.001
(11) AI medical tools should only be used if clinicians understand how AI decisions are made.	157 (78.9); 199	133 (83.1); 160	1.32 (0.77–2.25)	0.313
(6) Improving equity and inclusion should be the top priority when developing and deploying AI in healthcare.	149 (72.3); 206	119 (73.5); 162	1.06 (0.67–1.68)	0.809
(12) There is high risk for monopolistic behavior by private AI companies in the domain of healthcare.	148 (74.4); 199	133 (83.1); 160	1.7 (1.01–2.86)	0.047
(37) Most areas of healthcare can benefit from AI.	147 (78.6); 187	95 (63.3); 150	0.47 (0.29–0.76)	0.002
(24) All the funding allocated for AI is worthwhile if it can take over bureaucratic shores, such as note-taking, coding, and pattern finding.	135 (69.2); 195	107 (69); 155	0.99 (0.63–1.56)	0.968
(35) AI enhances medical decision making in situations of care rationing.	125 (65.4); 191	93 (61.6); 151	0.85 (0.54–1.32)	0.462
(36) AI will allow providers, clinicians, and staff, to focus on more top-of-license skill sets and activities.	125 (66.8); 187	83 (55.3); 150	0.61 (0.39–0.96)	0.031
(26) In the medical field it is problematic that machines lack contextual knowledge and ability to read social clues.	116 (60.1); 193	109 (71.2); 153	1.64 (1.04–2.59)	0.032
(27) It would be unethical not to use AI tools if they provide better decisions than medical doctors.	116 (60.1); 193	83 (54.2); 153	0.79 (0.51–1.21)	0.274
(40) If a medical doctor makes a mistake as a result of the advice from an AI tool, he/she should be considered liable.	90 (48.1); 187	97 (64.7); 150	1.97 (1.27–3.07)	0.003
(17) Appropriate informed consent is not possible if the medical doctor cannot explain to the patient how the AI medical device works.	89 (45.4); 196	99 (63.1); 157	2.05 (1.34–3.15)	0.001

^a^More than 60% of respondents agreed (value 4 or 5 at Likert scale) with the statement in at least one country.Numbers in brackets are sequential numbers of the questionnaire from Martinho et al. [25] (S1 Appendix)

Regarding remaining statements, Slovenian RTs are to lower extent (OR: 0.31; 95% CI: 0.19–0.49) conservative towards the promotion of AI. They agree to lower extent than Croatian RTs that most areas of healthcare can benefit from the use of AI (OR: 0.47; 95% CI: 0.29–0.76), and that providers, clinicians, and staff could focus on more complex tasks because of the use of AI (OR: 0.61; 95% CI: 0.39–0.96). On the other hand, they agree more with the statement that appropriate informed consent is not possible (OR: 2.05; 95% CI: 1.34–3.15), that medical doctor is responsible for the mistakes made by AI (OR: 1.97; 95% CI: 1.27–3.07), that there would be higher risk for the monopolistic behavior by AI companies (OR: 1.7; 95% CI: 1.01–2.86) and that AI tools lack of contextual knowledge might be problematic (OR: 1.64; 95% CI: 1.04–2.59).

**Table 3** includes remaining statements where the share of respondents that agree or strongly agree with the statement is lower than 60% in both countries. The highest share of respondents in both countries disagree with the use of AI on behalf of putting patients at risk. About half of the respondents in each country agree that doctors who use AI will replace those who do no and that patient-physician relationship will change. Although the share of respondents agreeing or strongly agreeing with the statement about the vulnerability of the computer systems to the cybersecurity threats is lower than 40% in both countries, this is the statement with the highest difference in opinion between countries (OR: 3.46; 95% CI: 1.99–6.02). Similar finding applies to the statement regarding liability of AI companies for medical errors (OR: 2.58; 95% CI: 1.27–5.25). Slovenian RTs also to higher extent agree, that big companies (influential and dominating companies that could monopolize every market branch) should not enter the health care space (OR: 1.65; 95% CI: 1.06–2.59), that AI might worsen problems within healthcare (OR: 1.83; 95% CI: 1.16–2.89), that AI products would not meet the expectations (OR: 1.73; 95% CI: 1.08–2.77) and that AI played vital role in COVID-19 pandemic (OR: 2.05; 95% CI: 1.18–3.57). Croatian RTs to higher extent than Slovenian RTs agree or strongly agree that only reliability is of interest to health professionals (OR: 0.38; 95% CI: 0.23–0.63) and that it is not difficult to operationalize clinical practice for a machine (OR: 0.35; 95% CI: 0.18–0.7).

**Table 3:** Remaining statements per country and results of univariate logistic regression (data shown as frequencies (percentages); number of respondents; OR = odds ratio; CI = confidence interval).

	Croatia (n = 214)	Slovenia (n = 165)	OR (95% CI)	P
(29) The mantra of the tech industry “fail fast and fix it later” is putting patients at risk and regulators are not doing enough to keep consumers safe.	108 (56); 193	84 (54.9); 153	0.96 (0.63–1.47)	0.844
(19) AI will not replace doctors, but doctors who use AI will replace doctors who do not.	102 (52); 196	74 (47.1); 157	0.82 (0.54–1.25)	0.36
(14) The patient-physician relationship will change dramatically once AI is fully deployed in health systems.	94 (47.2); 199	86 (53.8); 160	1.3 (0.86–1.97)	0.22
(15) Health professionals do not need to know how AI medical tools work but rather if they are reliable.	73 (36.7); 199	29 (18.1); 160	0.38 (0.23–0.63)	< 0.001
(5) AI is more likely to resolve rather than amplify inequalities in healthcare.	70 (32.7); 214	63 (38.2); 165	1.27 (0.83–1.94)	0.269
(4) Confidentiality, as defined today, has little use in a future where Healthcare relies heavily in AI.	66 (30.8); 214	55 (33.3); 165	1.12 (0.73–1.73)	0.606
(18) AI will decrease the autonomy and authority of medical doctors.	65 (33.2); 196	62 (39.5); 157	1.32 (0.85–2.04)	0.219
(2) Confidentiality should not constrain the implementation of AI in healthcare.	57 (26.6); 214	40 (24.2); 165	0.88 (0.55–1.41)	0.597
(22) Automation may work well in factories, but not in hospitals	57 (29.2); 195	60 (38.7); 155	1.53 (0.98–2.39)	0.063
(13) It is undesirable that big companies enter the health care space because they know little about medicine.	53 (26.6); 199	60 (37.5); 160	1.65 (1.06–2.59)	0.028
(16) Health professionals have always trusted black boxes (e.g. MRI) and it will not be different with AI	53 (27); 196	53 (33.8); 157	1.37 (0.87–2.17)	0.172
(21) AI will worsen problems in healthcare such as overtesting, overdiagnosis, and overtreatment.	50 (25.6); 195	60 (38.7); 155	1.83 (1.16–2.89)	0.009
(7) AI will increase discrimination based on predicted future medical problems.	45 (21.8); 206	50 (30.9); 162	1.6 (1–2.55)	0.051
(23) AI-based medical products won’t be able to match the hype.	45 (23.1); 195	53 (34.2); 155	1.73 (1.08–2.77)	0.022
(25) Doctors are not interested in learning about AI and computer science.	44 (22.6); 195	32 (20.6); 155	0.89 (0.53–1.49)	0.665
(34) Clinicians lack the time to learn how to use complex AI-based medical devices.	43 (22.5); 191	28 (18.5); 151	0.78 (0.46–1.33)	0.369
(20) If AI tools work well, hospitals should save money by hiring less highly skilled practitioners.	40 (20.4); 196	19 (12.1); 157	0.54 (0.3–0.97)	0.04
(38) It is not very difficult to operationalize clinical practice for a machine.	37 (19.8); 187	12 (8); 150	0.35 (0.18–0.7)	0.003
(31) Because AI systems are designed mainly to increase profit, in the future health systems will have more resources and provide better care.	32 (16.8); 191	19 (12.6); 151	0.72 (0.39–1.32)	0.284
(28) AI has already played a vital role in the COVID-19 pandemic.	26 (13.5); 193	37 (24.2); 153	2.05 (1.18–3.57)	0.011
(39) Medicine should never rely on AI because such computer systems are vulnerable to cybersecurity threats.	23 (12.3); 187	49 (32.7); 150	3.46 (1.99–6.02)	< 0.001
(1) Privacy should not be the highest priority in AI-based healthcare.	15 (7); 214	19 (11.5); 165	1.73 (0.85–3.51)	0.132
(10) For the sake of technology advancement AI companies should not be liable for medical errors.	13 (6.3); 206	24 (14.8); 162	2.58 (1.27–5.25)	0.009
ADDITIONAL STATEMENT				
AI is influencing decision-making in clinical and preclinical medicine	129 (69.4); 186	96 (64); 150	0.79 (0.50–1.24)	0.300

Numbers in brackets are sequential numbers of the questionnaire from Martinho et al. [25] (S1 Appendix)

The participants in both countries answered the statement that AI is influencing decision-making in clinical and preclinical medicine. Around two thirds of respondents in Slovenia (96 (64%); n = 150) and Croatia (129 (69.4%); n = 186) agreed or completely agreed with this statement. No statistically significant association between country and opinion on penetration of AI in medical decision making was found (OR: 0.79; 95% CI: 0.50–1.24).

**Fig 1:**
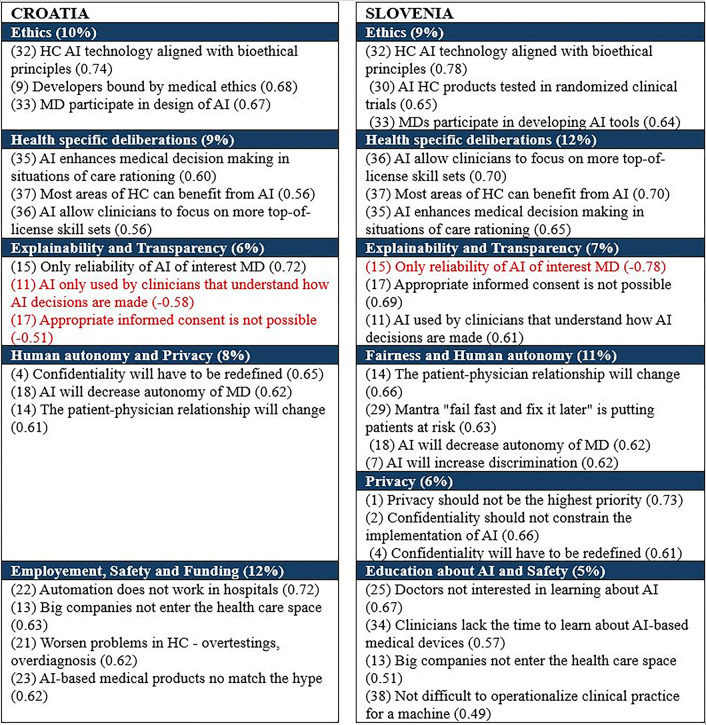
Perspectives about health AI–Croatia and Slovenia (three statements with the highest weights per component are shown; items with negative weight are written in red–opposite meaning; the numbers in brackets of the component name indicate % of explained variability HC = healthcare; numbers in brackets are sequential numbers of the questionnaire from Martinho et al. [25] (S1 Appendix)).

Five perspectives about Health AI in Croatia and six in Slovenia were identified by the principal component analysis (Fig 1). The analysis was performed separately for each country to capture the differences in perspectives. The components were named after the items with the highest weights. The name of the component was based on the content of the items. Martihno et al. [25] assigned each item in so-called AI clusters and the name of each component was based on the cluster name of the prevailing items with highest weights on the component. The first three components by country are identical–RTs perceptions are similar with regard of these AI issues in both countries (Fig 1). The remaining components differ between the countries, underlying the differences in perceptions between the RTs. Furthermore, the complexity of the AI ethics in medicine is higher in Slovenia than in Croatia.

## Discussion

In this study we surveyed Slovenian and Croatian RTs working at two Slovenian and four Croatian Schools of Medicine. The participants in both countries answered also the additional statement that AI is influencing decision-making in clinical and preclinical medicine. Approximately two thirds of respondents in Slovenia (96/150 (64%)) and Croatia (129/186 (69.4%)) agreed or completely agreed with this statement. There is no significant difference between countries. The RTs in Croatia and Slovenia showed some common and some different attitudes on AI use in healthcare when we compared the highest statements’ share (above 60%) or lowest share (below 60%). We examined the attitudes of RTs and five different ethical perspectives about healthcare AI use were identified among Croatian and six among Slovenian RTs. In the following paragraphs we will discuss each of these perspectives and examine the differences or similarities of perspectives between Croatian and Slovenian RTs.

### Ethical design perspective

Ethical principles are, since Nurnberg Code, the foundation for research and experiments where human beings are involved [30]. Moreover, it is imperative that such principles are consistently integrated within the context of new technological advancements, notably AI, as well [17, 31]. In that context our ethical design perspective confirms that statements on ethical dilemmas with the highest share and agreement per country (above 85%), analyzed with univariate logistic regression, did not show any statistical differences between two countries on two of the three statements relating to the ethical issues. The statements highlighted the importance of ethical principles that must be in line not only for RTs but also for AI developers. Croatian RTs focused more on AI developers to be bound by medical ethics, while Slovenian RTs focused more on attitude that AI healthcare products should be tested in randomized controlled trials (RCTs) which is in line with the Thomas Grote’s view that the clinical benefit of AI systems needs to be studied in clinical trials-particularly RCTs [18]. In both countries RTs strongly stressed that AI must be in line with ethics, an attitude that was recently discussed thoroughly in an article of Jeyarman et al. [31] and other recent studies [18, 32, 33]. The results align with those of Marthino et al., but they identify an additional attitude of privacy as a core ethical value, which is not evident from our perspective [25].

### Health-specific deliberation perspective

Statements with the highest agreement per country analyzed with univariate logistic regression showed significant differences in two of three statements in this perspective. Slovenian RTs agree to lower extent than Croatian RTs that most areas of healthcare can benefit from the AI use and that RTs could focus on more complex tasks because of the AI, while there are no differences in the statement that AI will enhance medical decision making in situations of care rationing. Despite lower agreement among Slovenian RTs those three statements are highly consistent with each other in both countries i. e. that AI will enhance medical decision making in situations of care rationing, allow providers, clinicians and staff to focus on more top-of license skill sets and activities together with believe that most area of healthcare can benefit from AI.

The three statements in health specific deliberation perspective emphasize again what are the expectations of RTs in Croatia and Slovenia as worldwide. The study by Katirai et al. [20] discussed the perspectives of patients and the public in Japan regarding the use of AI in medicine. They conducted a workshop, leading to the identification of 55 expectations and 52 concerns related to AI, which were categorized into 12 major themes. They found expectations for improvement of hospital administration, quality of care and patient experience, and positive changes in roles and relationships, and reductions in costs and disparities. However, were concerned about healthcare changes, loss of autonomy, risks of accountability, data management and disparities. Comparably in empirical study by Amann J. et al. [34] participants highlighted, besides all technical benefits of AI, the significance of relational aspects, expressing concerns about how AI influences roles, responsibilities, and the rights of patients to receive information and participate in decision-making processes. Moreover, not only ethical aspects but also sociological aspects are important in implementation of AI in healthcare which is comparable with the robotics care for older people [35]. These views are comprehensible since current medical AI systems do not have a capability of autonomous decision-making which thereby disqualify them as responsible agents. Consequently, the responsibility for AI’s actions should be firmly put on the human agent [36]. Our perspective aligns with the first one by Marthino et al. [25], which emphasized that AI is a useful tool allowing doctors to focus on top-of-license skills. However, they stress that, despite the positive outlook, medical doctors must remain in charge of both the medical decision process and the design process. These two attitudes are touched upon in our other perspectives, with no difference in results.

### Explainability and transparency perspective

Transparency and explainability are crucial and increasingly discussed attributes of AI systems, yet practical guidelines for defining these requirements are still subject of discussion for these are abstract terms that have a very concrete application [37]. Drobotowicz K. et al. in their empirical study extracted the following parameters for transparency: knowability of purposes, disclosing the nature and sources of the data they are utilized, providing easy explanations for users to understand, obtain individual insight into data management, human involvement in AI services [38]. In our third perspective statements with the highest or lowest agreement per country analyzed with univariate logistic regression showed significant differences in two of the three statements, i.e., only reliability of AI is of interest of RTs and appropriate informed consent is not possible while no differences were found in statement that AI is only used by clinicians that understand how AI decisions are made. The share of respondents that agree or strongly agree with the statements higher than 60% in both countries showed that Slovenian RTs agree more with the statement that appropriate informed consent is not possible. The share of respondents that agree or strongly agree with the statements lower than 60% in both countries showed that Croatian RTs to higher extent than Slovenian RTs agree or strongly agree that only reliability is of interest to RTs.

However, those three statements have rather diametrically opposite component weights in both countries. While high positive weight among Croatian RTs with variable “healthcare professionals do not need to know how AI medical tools work, only reliability of AI is interest of RTs” and high negative weight for variable “appropriate informed consent is not possible” diametrically opposed weights are obtained among Slovenian RTs.

These diametrically opposed meaning cannot be easily understood and put in the right context if we do not look at the second statement “appropriate informed consent is not possible” where diametrically opposed meaning of the statement is found again. Croatian RTs are sure that they can get appropriate informed consent because in their opinion there is no need to know how AI medical tools works. Slovenian RTs cannot explain how AI medical works and therefore appropriate informed consent is not possible. The third statement, indicating a Croatian RTs attitude with the component weight of -0.58 and a Slovenian RTs attitude of 0.61, supports the first two statements in both countries. This shows a consistency in the attitudes of RTs from both countries regarding the first statement. In other words, in Slovenia it is stressed that RTs need to know how AI works, how AI medical tools work, and without these two statements appropriate informed consent is not possible for them. In Croatia is stressed that RTs don’t need to know how AI works, how AI medical tools work, and because of that they believe that appropriate informed consent is possible for them. Numerous articles discuss the ethical challenge of explicability in AI, especially in medicine. In the context of our study, it is useful to highlight Adams’ proposal to include a ’principle of explicability’ alongside the traditional bioethics principles, and argues for explicability as an essential ethical consideration, enabling a bridge between technical demands and high-level ethical standards in AI use [11, 34, 39]. In their fourth perspective, Marthino et al. link explainability with education, while in our case, it is connected with the need for transparency. They state that explainability is a key value in the sense that, in order to reap the benefits of AI, physicians must understand and lead AI technological progress. They stress that physicians are interested in learning about AI and have time to learn how to use complex AI-based medical devices [25].

### Privacy perspective

The following perspectives differ in name and statements. Another concern expressed by RTs in both countries relates to Privacy. The use of AI in medicine is challenged by protection of data privacy as data can be impacted by manipulative goals [19, 40]. Privacy and confidentiality are the single most important statements with the highest weights per component seen only among Slovenian RTs and not among Croatian RTs, where privacy was joined with autonomy. In Slovenia RTs believes that confidentiality should not be the highest priority in AI-based healthcare or constrain the implementation of AI but that it will have to be redefined. Privacy is defined through confidentiality. In Croatia, however, confidentiality is closely connected with decrease of the autonomy of the RTs due to use of AI. They believe that it will decrease the autonomy, reshape the patient-physician relationship and therefore influence the very meaning of confidentiality. For comparison, in Marthino et. al. privacy is considered a core ethical and medical value in the perspective of ethics, i.e., it is linked with the ethical demands for AI implementation. From this perspective, AI will not increase discrimination and improving equity and inclusion is not mandated to be priority [25].

It is not clear why both statements have not so high weights in Slovenia, but it could be hypothesized. Firstly, that those applying AI in their work found that sharing data with AI medical systems cannot protect individual privacy and confidentiality. In that line confidentiality must be redefined. Secondly, according to paradigm of the evidence-based medicine, RTs involved in decision making process are not bound with classically patient-physician relationship. Thirdly, development of AI found RTs unprepared towards AI medical companies or they are excited about possibilities which AI offer while forgetting privacy and confidentiality. Indeed, the significance of these hypotheses is in line with the literature available [21, 22, 41].

### Autonomy and privacy/ fairness and autonomy perspective

RTs in Slovenia linked the autonomy with one of the core medical ethics principles–fairness (justice). They consider that the use of AI will change medical practice, and the ethical principle of fairness ensuring medical decisions are made fairly could be threatened. One of the major concerns expressed by Slovenian RTs are that AI will increase discrimination and that the mantra “fail fast and fix it later” will put patients at risk. In both countries RTs believe that AI will decrease autonomy and essentially change patient-physician relationship. In Croatia they think that confidentiality will change too, while in Slovenia more emphasis is put on concern about the increase of discrimination and risk for patient. These concerns are recognized in recent literature as well. Vearrier et al. [7] maintain that this relationship must be viewed as a physician-patient-machine (AI system) relationship. Despite all the benefits of AI systems, the physician’s roll is crucial for e.g. interpretation of data in the clinical context of an individual patient. In line with that a physician must advocate for patient confidentiality. In his study Kiener [41] notice, that in accordance with the current clinical practices, there are specific situations where risks must be disclosed to the patient. Otherwise, patient’s informed consent will be compromised or it will be breached the broader duty to alert them about possible adverse outcomes, what Slovenian RTs in our study apparently recognized. The issue of discrimination is essentially a matter of contextual knowledge, which AI systems lack, hence this requires systematic and ongoing intervention by the human. The fundamental issue on AI system implementation is the uncertainty of discrimination due to bias [15]. In Marthino et al. it is shown that big companies are not to be trusted, especially regarding medical data ownership. Their results show that AI will not increase discrimination based on predicted future problems if privacy becomes a key ethical value. Additionally, they emphasize that physicians must remain in charge in the medical decision process [25].

### Employment, safety and funding/ education about AI and safety perspective

The share of respondents agreeing or strongly agreeing with the statements regarding employment, safety and funding/education are well below 60% (lower than 40%, and down to 8%) in both countries. The countries also differed in perspectives. In the sixth perspective in Slovenia *Education and safety* RTs bring forward the lack of time or interest for learning about the AI tools on one hand and securing that big companies do not enter the AI medical market on the other addressed. There are similar concerns about the knowledge and skills of RTs regarding AI in similar surveys [23]. In Croatia one of the concerns is expressed in the fifth perspective on *Employment*, *safety and funding*. RTs expressed doubts that AI might not meet the expectations and that automate will not function in hospitals. It can lead to over-testing and over-diagnosis and hence worsening the problems in healthcare. As in Slovenia, also in Croatia the RTs discourage the entry of big companies in the health care space. Which can be considered justified since it concerns a relevant issue of liability and responsibility of RTs for AI systems failures [14, 28, 42]. Croatian RTs to higher extent agree that it is not difficult to operationalize clinical practice for a machine. Other authors such as Tahri et. al. [14] asserted that prior to undertaking such procedure, there is a necessary prerequisite for the humanization of AI, to guarantee that its design and implementation are in conformity with ethical principles. Nevertheless, these perspectives don’t seem to be very important for Croatian or Slovenian RTs. Marthino et al. emphasized that it is difficult to operationalize clinical practice for a machine and that physicians are interested in learning about AI. In their results, employment is linked with usefulness, showing that AI will not indiscriminately cause unemployment, and safety is associated with regulatory issues [25].

This study indicated the need for the further similar research, as it is the first in Slovenia and Croatia that comprehensively deals with the issue of RTs attitudes and perspectives on the AI use in medicine. These results provide a useful insight into the differences and similarities between the two neighboring countries. In spite the cultural and social similarities of these countries there are characteristic differences in views on AI use in medicine. There are some limitations of this study. It included only two countries, which is not enough if one wants to get a wider picture. Future research should involve RTs from a variety of countries to identify and compare differing attitudes and perspectives. In our research, we focused on RTs, i.e. highly educated health professionals who work in educational institutions, other profiles of health professionals are not covered in this study. This expanded scope would allow for a broader analysis of responses gathered from individuals within different kinds of medical institutions. Our findings could be of a great help in developing policies and legal regulations on AI use in medicine.

## Conclusion

This study represents the first investigation into the understanding of ethical attitudes and perspectives of AI use in medicine, among RTs at two Slovenian and four Croatian Schools of Medicine. Despite the interpretation presented in this article, much broader useful insights can be obtained from the results. However, not including in legal and ethical decision-making such real dilemmas and concrete issues faced by RTs AI use in medicine could lead to simplifications that RTs face the same dilemmas and issues to the same extend everywhere. Our results highlight the need for broaden this study pattern in order to facilitate understanding of the implications of AI use in medicine and set a solid data base for tackle ethical and legal issues.

There are some limitations of our study. The non-random sampling was used and the self-selection bias could be present in the study. Although the samples in the two countries were comparable regarding the demographical characteristics, the participants who decided to participate in the study could be different from those who did not. Even if applying random sampling, the research could lead to biased results due to non-response [43]. Regardless of the type of sampling, it would be expected, that participants were interested or concerned about the investigated topic, so the opinions and attitudes presented in this research are those of the concerned parties. It is expected that due to the AI developments the number of the concerned RTs will increase in the near future [4].

## Supporting information

S1 Appendix Original questionnaire items by Martinho et al. [25]. (DOCX)

S2 Appendix Adapted Slovenian version of Martinho et al. [25] questionnaire. (DOCX)

S3 Appendix Adapted Croatian version of Martinho et al. [25] questionnaire. (DOCX)

S1 Dataset Ethical considerations of health AI in Slovenia and Croatia dataset. (SAV)
