# Supplementary material for: Ethical attitudes and perspectives of AI use in medicine between Croatian and Slovenian faculty members of school of medicine: Cross-sectional study
Source: PLoS One. 2024 Dec 5;19(12):e0310599. doi: 10.1371/journal.pone.0310599 (PMC11620630; doi:10.1371/journal.pone.0310599)
Supplement: S1 Appendix — (DOCX) [file pone.0310599.s001.docx]

APPENDIX 1 Original questionnaire items by Martinho et al.

1. Privacy should not be the highest priority in AI-based Healthcare.

2. Confidentiality should not constrain the implementation of AI in Healthcare.

3. Without clear rules about data usage, storage, and anonymization, AI should never be used in Healthcare.

4. Confidentiality, as defined today, has little use in a future where Healthcare relies heavily in AI.

5. AI is more likely to resolve rather than amplify inequalities in healthcare.

6. Improving equity and inclusion should be the top priority when developing and deploying AI in healthcare.

7. AI will increase discrimination based on predicted future medical problems.

8. We should be conservative in promoting AI in healthcare because of the unresolved ethical issues.

9. AI developers must be bound by medical ethics.

10. For the sake of technology advancement AI companies should not be liable for medical errors.

11. AI medical tools should only be used if clinicians understand how AI decisions are made.

12. There is high risk for monopolistic behavior by private AI companies in the domain of Healthcare.

13. It is undesirable that big companies enter the health care space because they know little about Medicine.

14. The patient-physician relationship will change dramatically once AI is fully deployed in health systems.

15. Health professionals do not need to know how AI medical tools work but rather if they are reliable.

16. Health professionals have always trusted black boxes (e.g. MRI) and it will not be different with AI.

17. Appropriate informed consent is not possible if the medical doctor cannot explain to the patient how the AI medical device works.

18. AI will decrease the autonomy and authority of medical doctors.

19. AI will not replace doctors, but doctors who use AI will replace doctors who do not.

20. If AI tools work well, Hospitals should save money by hiring less highly skilled practitioners.

21. AI will worsen problems in healthcare such as overtesting, overdiagnosis, and overtreatment.

22. Automation may work well in factories, but not in Hospitals.

23. AI-based medical products won’t be able to match the hype.

24. All the funding allocated for AI is worthwhile if it can take over bureaucratic shores, such as note-taking, coding, and patternfinding.

25. Doctors are not interested in learning about AI and Computer Science.

26. In the medical field it is problematic that machines lack contextual knowledge and ability to read social clues.

27. It would be unethical not to use AI tools if they provide better decisions than medical doctors.

28. AI has already played a vital role in the COVID-19 pandemic.

29. The mantra of the tech industry “fail fast and fix it later” is putting patients at risk and regulators are not doing enough to keep

consumers safe.

30. AI healthcare products must be tested in randomized clinical trials, which is the strongest source of medical evidence.

31. Because AI systems are designed mainly to increase profit, in the future health systems will have more resources and provide better care.

32. Healthcare AI technology must be aligned with bioethical principles.

33. Medical doctors must participate in the design process of AI for Healthcare.

34. Clinicians lack the time to learn how to use complex AI-based medical devices.

35. AI enhances medical decision making in situations of care rationing.

36. AI will allow providers, clinicians, and staff, to focus on more topof-license skill sets and activities.

37. Most areas of healthcare can benefit from AI.
38. It is not very difficult to operationalize clinical practice for a machine.
39. Medicine should never rely on AI because such computer systems are vulnerable to cybersecurity threats.
40. If a medical doctor makes a mistake as a result of the advice from an AI tool, he/she should be considered liable.
