# Supplementary material for: Ethical attitudes and perspectives of AI use in medicine between Croatian and Slovenian faculty members of school of medicine: Cross-sectional study
Source: PLoS One. 2024 Dec 5;19(12):e0310599. doi: 10.1371/journal.pone.0310599 (PMC11620630; doi:10.1371/journal.pone.0310599)
Supplement: S2 Appendix — (DOCX) [file pone.0310599.s002.docx]

**Etični vidiki umetne inteligence v medicini**

*Spoštovani!
Sistemi umetne inteligence v medicini danes pomagajo pri kliničnem in raziskovalnem delu, pri postavljanju diagnoz in podpori pri kliničnih odločitvah v najrazličnejših vejah medicine ter pri razvijanju zdravil in cepiv.****Umetna inteligenca pomeni računalniške sisteme, ki iz okolja pridobivajo podatke, na podlagi katerih lahko nato avtonomno delujejo na človeku podoben način (se učijo in odločajo, sklepajo, napovedujejo, prepoznavajo vzorce).*** *Prosimo vas za sodelovanje v anketi o etičnih vidikih uporabe umetne inteligence v medicini in zdravstvu. Anketa obsega 4 krat po 10 trditev. Prosimo vas, da svoje stališče do vsake trditve ocenite na lestvici od "sploh se ne strinjam" do "popolnoma se strinjam". Prosimo vas tudi, da oceno podate pri vseh trditvah.
Anketa je anonimna, njeno izpolnjevanje pa traja približno 10 minut.*

*Avtorji raziskave:* Eva Flajnik, študentka medicine,  prof. dr. Štefan Grosek, dr. med., višji svetnik, dr. Stjepan Štivić, mag. fil., univ. bacc. filozofije in religiologije, doc. dr. Vanja Erčulj, dipl. psih., Jaro Lajovic, dr. med., Jonas Miklavčič, mag. fil. in dipl. teol. štud.

1. **Prosimo vas, da 5-stopenjski lestvici strinjanja označite, v kolikšni meri se strinjate z naslednjimi trditvami.**

| **TRDITVE** | Sploh se ne strinjam | Ne strinjam se | Niti - niti | Strinjam se | Popolnoma se strinjam |
| --- | --- | --- | --- | --- | --- |
| 1. V zdravstvu, ki temelji na umetni inteligenci, zasebnost ne bi smela biti najvišja prioriteta. |  |  |  |  |  |
| 1. Zaupnost ne bi smela omejevati uvajanja umetne inteligence v zdravstvo. |  |  |  |  |  |
| 1. Umetne inteligence v zdravstvu ne bi smeli uporabljati brez jasnih pravil o rabi, shranjevanju in anonimizaciji podatkov. |  |  |  |  |  |
| 1. V prihodnosti, ko bo zdravstvo močno slonelo na umetni inteligenci, bo današnje razumevanje zaupnosti slabo uporabno. |  |  |  |  |  |
| 1. Umetna inteligenca bo neenakost v zdravstvu bolj verjetno odpravila kot dodatno povečala. |  |  |  |  |  |
| 1. Glavna prioriteta pri razvoju in uporabi umetne inteligence v zdravstvu bi moralo biti izboljšanje enakosti in vključenosti. |  |  |  |  |  |
| 1. Umetna inteligenca za napovedovanje zdravstvenih težav v prihodnosti, bo povečala diskriminacijo. |  |  |  |  |  |
| 1. Zaradi nerešenih etičnih vprašanj moramo biti pri spodbujanju rabe umetne inteligence v zdravstvu zadržani. |  |  |  |  |  |
| 1. Razvijalce umetne inteligence mora zavezovati medicinska etika. |  |  |  |  |  |
| 1. V korist tehnološkega napredka podjetja s področja umetne inteligence ne bi smela biti odgovorna za napake pri zdravljenju. |  |  |  |  |  |
| 1. Medicinsko opremo, ki temelji na umetni inteligenci, bi smeli uporabljati le, če zdravniki razumejo, kako umetna inteligenca sprejema odločitve. |  |  |  |  |  |
| 1. V zdravstvu obstaja veliko tveganje za monopolno obnašanje zasebnih podjetij s področja umetne inteligence. |  |  |  |  |  |
| 1. Ni zaželeno, da velika podjetja vstopajo na področje zdravstva, ker o medicini vedo le malo. |  |  |  |  |  |
| 1. Ko se bo umetna inteligenca v zdravstvu povsem uveljavila, se bo odnos med zdravnikom in bolnikom močno spremenil. |  |  |  |  |  |
| 1. Zdravstvenim delavcem ni treba vedeti, kako medicinska oprema z umetno inteligenco deluje, temveč predvsem, ali je zanesljiva. |  |  |  |  |  |
| 1. Zdravstveni delavci so vedno zaupali črnim škatlam (npr. magnetni resonanci) in z umetno inteligenco ne bo nič drugače. |  |  |  |  |  |
| 1. Ustrezna privolitev po poučitvi ni mogoča, če zdravnik bolniku ne more razložiti, kako deluje medicinski pripomoček z umetno inteligenco. |  |  |  |  |  |
| 1. Umetna inteligenca bo zmanjšala avtonomijo in avtoriteto zdravnikov. |  |  |  |  |  |
| 1. Umetna inteligenca ne bo nadomestila zdravnikov, bodo pa zdravniki, ki uporabljajo umetno inteligenco, nadomestili tiste zdravnike, ki je ne. |  |  |  |  |  |
| 1. Če bodo sistemi umetne inteligence dobro delovali, bodo bolnišnice prihranile denar, saj bodo lahko najemale manj vešče strokovnjake. |  |  |  |  |  |
| 1. Umetna inteligenca bo v zdravstvu še povečala težave, kot so prekomerno testiranje, prekomerno diagnosticiranje in prekomerno zdravljenje. |  |  |  |  |  |
| 1. Avtomatizacija lahko dobro deluje v tovarnah, ne pa tudi v bolnišnicah. |  |  |  |  |  |
| 1. Medicinska oprema z umetno inteligenco ne bo mogla izpolniti velikih pričakovanj. |  |  |  |  |  |
| 1. Vsa sredstva, namenjena umetni inteligenci, so upravičena, če ta lahko prevzame birokratske naloge, kot so zapisovanje, kodiranje in iskanje vzorcev. |  |  |  |  |  |
| 1. Zdravnikov ne zanima učenje o umetni inteligenci in računalništvu. |  |  |  |  |  |
| 1. Na medicinskem področju je problematično, da računalniki nimajo kontekstualnega znanja in sposobnosti prepoznavanja socialnih signalov. |  |  |  |  |  |
| 1. Neetično bi bilo, da ne bi uporabljali sistemov umetne inteligence, če zagotavljajo boljše odločitve kot zdravniki. |  |  |  |  |  |
| 1. Umetna inteligenca je že odigrala pomembno vlogo pri pandemiji COVID-19. |  |  |  |  |  |
| 1. Mantra tehnološke industrije "neuspeh hitro, popravljanje pozneje" ogroža bolnike, regulatorji pa ne storijo dovolj za zagotovitev varnosti potrošnikov. |  |  |  |  |  |
| 1. Sisteme umetne inteligence, namenjene zdravstvu, je treba testirati v randomiziranih kliničnih preskušanjih, ki so najpomembnejši vir medicinskih dokazov. |  |  |  |  |  |
| 1. Ker so sistemi umetne inteligence zasnovani predvsem za povečanje dobička, bodo imeli zdravstveni sistemi v prihodnje več sredstev in bodo zagotavljali boljšo oskrbo. |  |  |  |  |  |
| 1. Zdravstvena tehnologija z umetno inteligenco mora biti usklajena z načeli bioetike. |  |  |  |  |  |
| 1. Zdravniki morajo sodelovati v načrtovanju umetne inteligence za zdravstvo. |  |  |  |  |  |
| 1. Zdravniki nimajo časa, da bi se učili uporabljati zapletene medicinske naprave, ki temeljijo na umetni inteligenci. |  |  |  |  |  |
| 1. Umetna inteligenca izboljšuje medicinsko odločanje v okoliščinah racionirane/omejene zdravstvene oskrbe. |  |  |  |  |  |
| 1. Umetna inteligenca bo izvajalcem, zdravnikom in osebju omogočila, da se bodo posvetili le veščinam in dejavnostim na najvišji ravni. |  |  |  |  |  |
| 1. Umetna inteligenca lahko koristi večini področij zdravstva. |  |  |  |  |  |
| 1. Klinične prakse ni prav težko operacionalizirati/pretvoriti za računalnike. |  |  |  |  |  |
| 1. Medicina se nikoli ne bi smela zanašati na umetno inteligenco, kajti takšni računalniški sistemi so izpostavljeni kibernetskim varnostnim grožnjam. |  |  |  |  |  |
| 1. Če zdravnik naredi napako zaradi nasveta sistema umetne inteligence, mora biti zanjo odgovoren. |  |  |  |  |  |

1. **Prosimo, da svoje strinjanje oziroma nestrinjanje opredelite še za spodnjo trditev.**

|  | Sploh se ne strinjam | Ne strinjam se | Niti - niti | Strinjam se | Popolnoma se strinjam |
| --- | --- | --- | --- | --- | --- |
| Umetna inteligenca prodira v odločanje o zdravljenju v klinični in predklinični medicini. |  |  |  |  |  |

**DEMOGRAFSKI PODATKI**

1. Starost: ............let
2. Spol: M

Ž

1. Trajanje delovne dobe:.............let
2. Delovno mesto in/ali specialnost (obkrožite odgovor):
3. Predklinična dejavnost
4. Klinična dejavnost
5. Drugo
