# Supplementary material for: Ethical attitudes and perspectives of AI use in medicine between Croatian and Slovenian faculty members of school of medicine: Cross-sectional study
Source: PLoS One. 2024 Dec 5;19(12):e0310599. doi: 10.1371/journal.pone.0310599 (PMC11620630; doi:10.1371/journal.pone.0310599)
Supplement: S3 Appendix — (DOCX) [file pone.0310599.s003.docx]

**Etičke dileme kod korištenja umjetne inteligencije u medicini**

*Poštovani,*

*Sustavi umjetne inteligencije u medicini danas pomažu u kliničkom i istraživačkom radu, u postavljanju dijagnoze i potpori u kliničkom odlučivanju u najrazličitijim granama medicine i u razvijanju lijekova i cjepiva.*

***Pod pojmom umjetna inteligencija se misli na računalne sustave, koji iz okoline pridobivaju podatke, na podlozi kojih potom mogu autonomno djelovati na čovjeku sličan način (uče i donose odluke, zaključuju, predviđaju, prepoznaju obrasce).***

*Molimo vas za sudjelovanje u anketnom upitniku o etičkim vidicima upotrebe umjetne inteligencije u medicini i zdravstvu. Upitnik sadrži 4 puta po 10 tvrdnji. Molimo vas, da svoj stav o svakoj tvrdnji ocijenite na ljestvici od „u potpunosti se ne slažem“ do „u potpunosti se slažem“. Ujedno vas molimo, da ocijenite svaku tvrdnju.*

*Anketni upitnik je anoniman, njegovo ispunjavanje traje približno 10 minuta. Anketa je anonimna i potrebno je oko 10 minuta. Odlukom o otvaranju i odgovaranju na upitnik potvrđujete da ste suglasni da se vaši od*

*govori anonimno koriste u obradi podataka.*

Upitnik je uporabljen u svom izvornom obliku u međunarodnom istraživanju: Martinho i sur. *A healthy debate: Exploring the views of medical doctors on the ethics of artificial intelligence* (Martinho A, Kroesen M, Chorus C. A healthy debate: Exploring the views of medical doctors on the ethics of artificial intelligence. Artif Intell Med [Internet]. 2021 Oct 12 [cited 2023 Feb 7];121:102190. Available from: https://pubmed.ncbi.nlm.nih.gov/34763805/ DOI: 10.1016/j.artmed.2021.102190.

Dana 5. prosinca 2022. dobili smo suglasnost autora navedenog upitnika (koji sadrži 40 tvrdnji) za provedbu istoga u našem istraživanju.

Istraživanje provodimo u suradnji: Medicinski fakulteti u Ljubljani i Mariboru, te Medicinski fakulteti u Zagrebu, Osijeku, Rijeci i Splitu.

**U istraživanju sudjeluju:**

Slovenija: Prof. dr. sc. Štefan Grosek, doc. dr. sc. Vanja Erčulj, dr. sc. Stjepan Štivić, Eva Flajnik, Jonas Miklavčič, Jaro Lajovic, mag. sc. Mirjana Mikšić

Hrvatska: Prof. dr. sc. Ana Borovečki, dr. sc. Marko Ćurković, prof. dr. sc. Ana Marušić, dr. sc. Ivan Buljan, dr. sc. Antonija Mijatović, prof. dr. sc. Suzana Mimica Matanović i prof. dr. sc. Kristina Lah Tomulić

1. **Molimo Vas, da naznačite u kojoj se mjeri slažete sa sljedećim izjavama na skali slaganja od 5 stupnjeva.**

| **Tvrdnje** | **U potpunosti se ne slažem** | **Ne slažem se** | **Niti se slažem, niti se ne slažem** | **Slažem se** | **U potpunosti se slažem** |
| --- | --- | --- | --- | --- | --- |
| 1.U zdravstvu temeljenom na umjetnoj inteligenciji privatnost ne bi trebala biti prioritet. |  |  |  |  |  |
| 2.Povjerljivost ne bi trebala ograničavati uvođenje umjetne inteligencije u zdravstvo. |  |  |  |  |  |
| 3.Umjetnu inteligenciju nikada ne bi trebalo koristiti u zdravstvu bez jasnih pravila o uporabi, pohrani i anonimizaciji podataka. |  |  |  |  |  |
| 4.Povjerljivost, kako ju danas definiramo, u budućnosti neće biti primjenjiva u zdravstvu koje se snažno oslanja na umjetnu inteligenciju. |  |  |  |  |  |
| 5.Vjerojatnije je da će umjetna inteligencija riješiti nejednakosti u zdravstvu, nego ih povećati. |  |  |  |  |  |
| 6.U razvoju i primjeni umjetne inteligencije u zdravstvu prioritet bi trebao biti poboljšanje pravičnosti i uključivosti. |  |  |  |  |  |
| 7.Umjetna inteligencija korištena u svrhu predviđanja budućih zdravstvenih problema će povećati diskriminaciju. |  |  |  |  |  |
| 8.Trebali bismo oprezno pristupiti promicanju umjetne inteligencije, jer postoje otvorena etička pitanja. |  |  |  |  |  |
| 9.Razvojni programeri umjetne inteligencije trebali bi se pridržavati načela medicinske etike. |  |  |  |  |  |
| 10.Radi potrebe unaprjeđenja tehnologije, tvrtke koje razvijaju umjetnu inteligenciju ne bi trebale odgovarati za medicinske pogreške. |  |  |  |  |  |
| 11.Medicinska rješenja koja koriste umjetnu inteligenciju trebala bi se upotrebljavati isključivo ako kliničari razumiju kako umjetna inteligencija donosi odluke. |  |  |  |  |  |
| 12.U zdravstvu postoji visoki rizik od monopolističkoga ponašanja privatnih tvrtki koje razvijaju umjetnu inteligenciju. |  |  |  |  |  |
| 13.Nije poželjno da velike tvrtke ulaze u područje zdravstva jer o medicini znaju malo. |  |  |  |  |  |
| 14.Odnos između liječnika i pacijenta dramatično će se promijeniti kada se umjetna inteligencija počne u potpunosti primjenjivati u zdravstvu. |  |  |  |  |  |
| 15.Zdravstveni djelatnici ne moraju znati kako rade medicinska rješenja koja koriste umjetnu inteligenciju, nego prije svega jesu li pouzdana. |  |  |  |  |  |
| 16.Zdravstveni djelatnici su uvijek imali povjerenja prema crnim kutijama (npr. magnetna rezonancija), a ni s umjetnom inteligencijom ne će biti drugačije. |  |  |  |  |  |
| 17.Odgovarajući informirani pristanak nije moguć ako liječnik ne može pacijentu objasniti kako radi medicinski uređaj koji koristi umjetnu inteligenciju. |  |  |  |  |  |
| 18.Umjetna inteligencija će smanjiti autonomiju i autoritet liječnika. |  |  |  |  |  |
| 19.Umjetna inteligencija ne će zamijeniti liječnike, nego će liječnici koji koriste umjetnu inteligenciju zamijeniti one koji ju ne koriste. |  |  |  |  |  |
| 20.Ako sustavi umjetne inteligencije budu dobro radili, bolnice će uštedjeti novac upošljavanjem manje osposobljenih liječnika. |  |  |  |  |  |
| 21.Umjetna inteligencija će povećati probleme u zdravstvu poput prekomjernog testiranja, prekomjernog dijagnosticiranja i prekomjernog liječenja. |  |  |  |  |  |
| 22.Automatizacija možda dobro djeluje u tvornicama, ali ne i u bolnicama. |  |  |  |  |  |
| 23.Medicinski proizvodi koji koriste umjetnu inteligenciju ne će biti u stanju ispuniti velika očekivanja. |  |  |  |  |  |
| 24.Sva sredstava uložena u umjetnu inteligenciju isplativa su ako će moći preuzeti birokratske zadaće kao što je uzimanje bilježaka, kodiranje i traženje obrazaca. |  |  |  |  |  |
| 25.Liječnike ne zanima učenje o umjetnoj inteligenciji i računarstvu. |  |  |  |  |  |
| 26.U području medicine problematično je to što strojevi nemaju kontekstualno znanje ni sposobnost prepoznavanja društvenih obrazaca. |  |  |  |  |  |
| 27.Bilo bi neetično ne koristiti sustave umjetne inteligencije ako oni donose bolje odluke od liječnika. |  |  |  |  |  |
| 28.Umjetna inteligencija je već odigrala ključnu ulogu u pandemiji COVID-19. |  |  |  |  |  |
| 29.Mantra tehnološke industrije „pogriješi brzo i popravi kasnije” ugrožava pacijente, a regulativna tijela ne čine dovoljno da ih zaštite. |  |  |  |  |  |
| 30.Sustavi umjetne inteligencije namijenjeni zdravstvu moraju se ispitati u randomiziranim kliničkim ispitivanjima, jer je to najviša razina medicinskih dokaza. |  |  |  |  |  |
| 31.S obzirom na to da su sustavi umjetne inteligencije napravljeni prvenstveno radi povećanja dobiti, zdravstveni sustavi će u budućnosti imati više sredstava i pružat će bolju skrb. |  |  |  |  |  |
| 32.Zdravstvena tehnologija koja koristi umjetnu inteligenciju mora biti usklađena s načelima bioetike. |  |  |  |  |  |
| 33.Liječnici moraju surađivati u postupku oblikovanja umjetne inteligencije namijenjene zdravstvu. |  |  |  |  |  |
| 34.Kliničari nemaju vremena učiti kako se koristiti složenim medicinskim uređajima koji koriste umjetnu inteligenciju. |  |  |  |  |  |
| 35.Umjetna inteligencija poboljšava donošenje medicinskih odluka u okolnostima ograničene dostupnosti primjerene (racionirane) zdravstvene skrbi. |  |  |  |  |  |
| 36.Umjetna inteligencija će omogućiti pružateljima skrbi, kliničarima i osoblju, da se usredotoče na profesionalne vještine i zadatke višega stupnja. |  |  |  |  |  |
| 37.Umjetna inteligencija može biti od koristi u većini područja u zdravstvu. |  |  |  |  |  |
| 38.Kliničku praksu nije pretjerano teško prilagoditi (operacionalizirati) za stroj. |  |  |  |  |  |
| 39.Medicina se uopće ne bi trebala oslanjati na umjetnu inteligenciju, jer su takvi računalni sustavi izloženi kibersigurnosnim prijetnjama. |  |  |  |  |  |
| 40.Ako liječnik učini pogrešku temeljem savjeta sustava umjetne inteligencije, liječnika se treba smatrati odgovornim. |  |  |  |  |  |

*Umjetna inteligencija prodire u donošenje odluka o liječenju u kliničkoj i pretkliničkoj medicini.

| Uopće se ne slažem | Ne slažem se | Niti se slažem niti se ne slažem | Slažem se | U potpunosti se slažem |
| --- | --- | --- | --- | --- |

1. Dob: ...............godina
2. Spol: M

Ž

1. **Radni staž:............godina**

**Radno mjesto i/ili specijalnost (zaokružite odgovor):**

- Pretklinička djelatnost
- Klinička djelatnost
- Drugo

**Medicinski fakultet (zaokružite odgovor):**

- Osijek
- Rijeka
- Split
- Zagreb
